# The Relationship Between Breast Milk Components and the Infant Gut Microbiota

**DOI:** 10.3389/fnut.2021.629740

**Published:** 2021-03-22

**Authors:** Gaëlle Boudry, Elise Charton, Isabell Le Huerou-Luron, Stéphanie Ferret-Bernard, Sophie Le Gall, Sergine Even, Sophie Blat

**Affiliations:** ^1^Institut NuMeCan, INRAE, INSERM, Univ Rennes, Saint-Gilles, France; ^2^UMR STLO INRAE, Institut Agro, Rennes, France; ^3^INRAE, UR BIA, Nantes, France; ^4^INRAE, BIBS facility, Nantes, France

**Keywords:** milk oligosaccharides, milk bacteria, milk lipids, gut microbiota, maternal diet

## Abstract

The assembly of the newborn's gut microbiota during the first months of life is an orchestrated process resulting in specialized microbial ecosystems in the different gut compartments. This process is highly dependent upon environmental factors, and many evidences suggest that early bacterial gut colonization has long-term consequences on host digestive and immune homeostasis but also metabolism and behavior. The early life period is therefore a “window of opportunity” to program health through microbiota modulation. However, the implementation of this promising strategy requires an in-depth understanding of the mechanisms governing gut microbiota assembly. Breastfeeding has been associated with a healthy microbiota in infants. Human milk is a complex food matrix, with numerous components that potentially influence the infant microbiota composition, either by enhancing specific bacteria growth or by limiting the growth of others. The objective of this review is to describe human milk composition and to discuss the established or purported roles of human milk components upon gut microbiota establishment. Finally, the impact of maternal diet on human milk composition is reviewed to assess how maternal diet could be a simple and efficient approach to shape the infant gut microbiota.

## Introduction

Under normal circumstances, the gut microbiota has a symbiotic relationship with the host. However, many chronic human diseases, including obesity, diabetes, cirrhosis, rheumatoid arthritis, and inflammatory bowel disease, have been associated with alterations in gut microbial communities (1). The infant gut microbiota is shaped in the first thousand days of life (2). Growing body of evidence revealed that altered neonatal colonization and disturbed interactions between the gut microbes and the host during the neonatal period could affect health later in life (3). The microbial colonization process is an orchestrated phenomenon resulting in specialized microbial ecosystems in the different gut compartments. However, this colonization process can be influenced by numerous environmental factors (4). One of the preponderant factors is neonatal diet, and it is largely accepted that human milk (HM) is the optimal diet that stimulates the most adequate microbiota development for the infant. Contrary to what is recommended by the World Health Organization (5), HM is provided for <6 months for a large percentage of children in Western countries (6). However, even if breastfeeding does not cover the whole microbiota maturation period, breastfeeding status was more associated with the infant gut microbiota composition than solid food introduction in a cohort of 323 healthy infants (7). The importance of HM upon shaping of the infant gut microbiota is also highlighted by the fact that cessation of breastfeeding, rather than introduction of solid food, was required for maturation into an adult-like microbiota in a cohort of 100 Swedish infants (8).

HM is a complex biofluid that provides all the nutrients required to promote infant growth. Beyond HM nutritional properties, the beneficial properties of breastfeeding on risk reduction of infant disease are well-recognized. HM is composed of a large diversity of components classified by their size into two main groups: macronutrients (fat, proteins, and carbohydrates) and micronutrients (vitamins, minerals, etc.), both dispersed between aqueous and colloidal phases (9, 10). HM also contains many bacterial species (11), immunomodulatory components (12), and hormones (13). HM composition is influenced by many factors such as the lactation period, with a different composition whether colostrum (first 48–72 h), transitional milk, and mature milk (from the second week of lactation until the end of lactation) are considered (14). Length of gestation, time of the day, phase of the nursing process (foremilk and hindmilk), and geographical and/or genetic female background also influence HM composition (15–18). Maternal diet also impacts HM composition, mainly fat composition as well as immunomodulatory components and bacterial species, whereas carbohydrate and protein contents seem less sensitive to the maternal diet (19, 20). The beneficial role of HM on gut microbiota development has been mainly attributed to the presence of oligosaccharides (21). However, the contribution of other HM components is also supported by the literature data. Although most of these data are associations between HM components and the infant gut microbiota or are derived from *in vitro* studies, thus not showing causal relationships, they are sometimes supported by human and animal model data. Moreover, most of the HM compounds, except milk oligosaccharides, are likely to be digested and absorbed before reaching the colon. However, a small fraction of the nutrients escapes small intestinal digestion. The amount of total lipids and proteins that reaches the colon under physiological conditions in adults has been evaluated to be between 5 and 8 g per day for dietary lipids (22) and 2–5 g per day for dietary proteins (23). In infants, data are scarce, but piglet studies revealed the presence of small fractions of dietary di- and monoacylglycerides and polar lipids as well as dairy proteins, either intact or as peptides in the ileum of piglets (24). Thus, a role of these HM compounds' fraction on infant colonic microbiota can be purported. The objective of this review is therefore to present the available data suggesting a role of various HM components on shaping the infant gut microbiota. The second objective is to evaluate how maternal diet, through its effect on these HM components, could be a potential leverage to orientate the infant gut microbiota and ensure optimal health.

## Human Milk Composition

### Macronutrients

**Figure 1:**
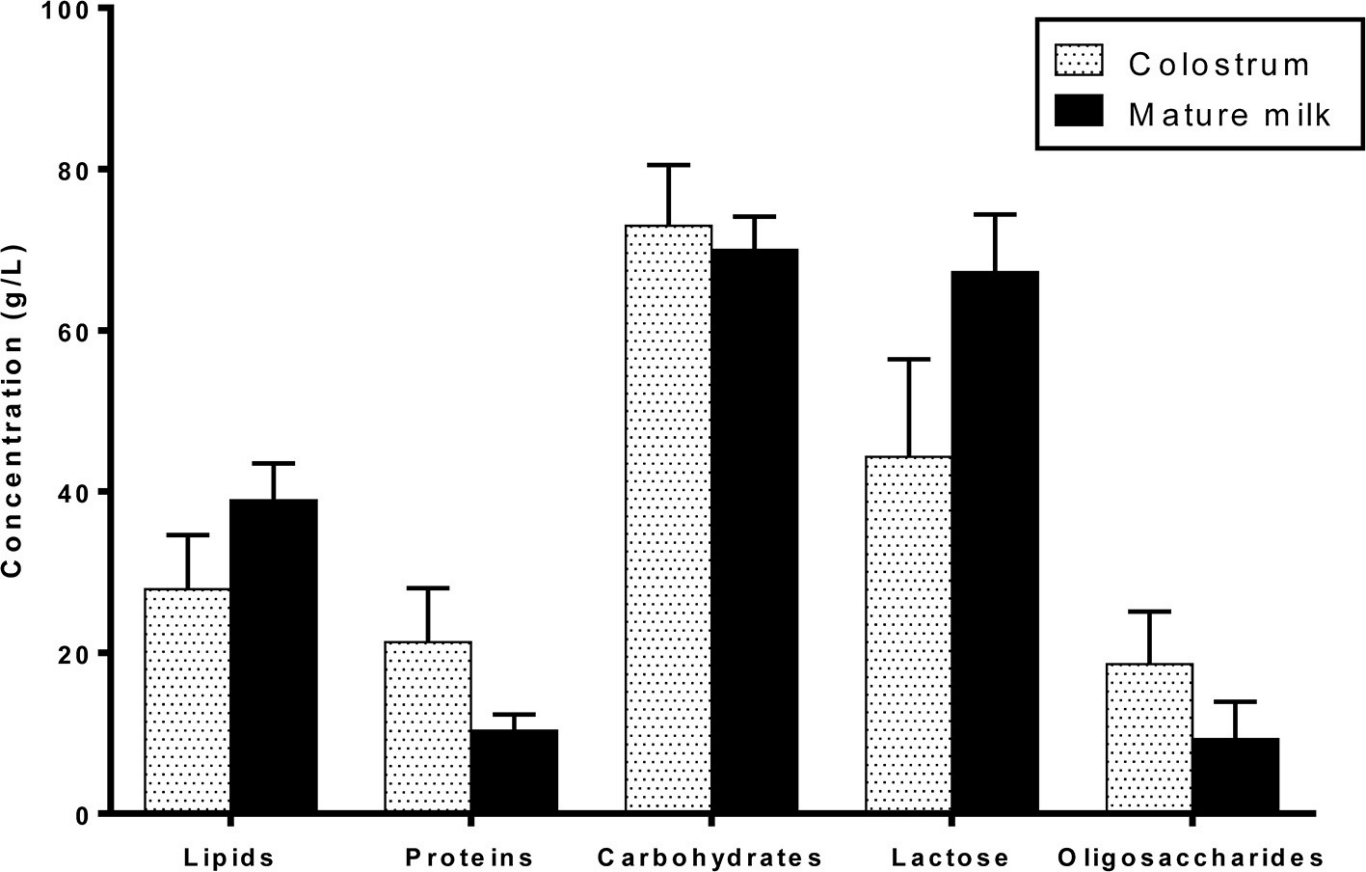
Macronutrient composition of colostrum and mature term human milk.

HM macronutrients are composed of lipids, proteins, and carbohydrates. Their concentrations vary over the lactation period from colostrum to mature milk (Figure 1): lipids and lactose content increase while proteins and oligosaccharide content decrease mainly during the first month of lactation and very slightly during mature milk stage (25, 26). Macronutrient concentration and type, especially lipid and protein contents, are slightly variable due to the multiple factors impacting HM composition including lactation time, feeding time, or mother's diet for example (15).

#### Lipids

Lipids are the main macronutrient in terms of energy. They represent 40–60% of energy in mature milk (26–28) and are the second most abundant macronutrient. They provide essential nutrients like polyunsaturated fatty acids and complex lipids (29). Lipids consist of 98% of triacylglycerides; the remaining is composed of diacylglycerides, monoacylglycerides, free fatty acids, phospholipids, and cholesterol. HM contains more than 200 fatty acids present in different concentrations (18). Oleic, palmitic, and linoleic acids, respectively, located in sn-1, sn-2, and sn-3 positions of triacylglycerides (30), are the highest concentrated ones. HM lipids are endogenously produced by the mammary gland or derived from maternal plasma (31). HM fat is packaged into lipid globules with triacylglycerols found in the core and surrounded by a bulk of phospholipids (32). The diameter of milk fat globules varies from 1 to 10 μm with an average diameter of 4 μm in mature milk (33).

Lipid content and composition are affected by many parameters: (i) feed phase (foremilk or hindmilk), respectively, 32 and 56 g/L (34, 35); (ii) lactation stage, lipid content being greater in mature milk than in colostrum; and (iii) maternal diet, which does not impact lipid content but impacts fatty acid profile and particularly that of the long-chain polyunsaturated fatty acids (17, 19, 31).

#### Proteins and Nitrogen

HM contains a wide range of proteins classified into three major classes: whey proteins, caseins, and mucins. Whey protein is the major fraction of HM proteins and is mostly represented by α-lactalbumin, lactoferrin (LF), lysozyme, and secretory immunoglobulin A (SIgA) (see below for their specific immunity role). α-Lactalbumin is involved in lactose synthesis (36) and has an amino acid composition similar to the amino acid requirement of the infant (37). Casein fraction includes α-, β-, and κ-casein with a predominance of β- and κ-casein (12). They are the main sources of minerals for the infant, including calcium and phosphorus. Casein function is mainly nutritive (38). The whey protein:casein ratio varies with lactation stage from 90:10 in colostrum to 60:40 in mature milk (12, 37, 39). Moreover, total protein level decreases from the first to sixth month of lactation (18). Protein content contributes to the infant growth, particularly by providing essential amino acids, and participates in immune protection and gut development (25, 39). HM also contains mucins, which belong to the glycoprotein family and are located in the milk fat globule membrane (25). Mucins 1 and 4 are the most studied mucins. Finally, 600 peptides have been recently identified in HM, which have an array of bioactive functions, including antimicrobial activity (40, 41).

##### Proteins and Peptides With Immunomodulatory and Growth Promotion Activities

LF (20% of total proteins) is found at high concentrations (5 g/L) in colostrum compared with mature milk (3 g/L). LF is a multifunctional protein of the transferrin family and is widely represented in various secretory fluids, like HM (42). LF has both bacteriostatic and bactericidal activities, limiting the growth of several pathogens and killing others. SIgAs in HM are one of the most abundant Igs (43) and the predominant antibody-mediated immune protection in mucosal surfaces of suckling infants. SIgA concentration is high in colostrum (5 g/L) and decreases in mature milk (1.5 g/L) (44). SIgAs provide specific protection against pathogens to which the mother has been previously exposed, via the entero-mammary pathway (45, 46). Activated B cells differentiate into plasma cells that synthesize high-affinity dimeric IgA in the mammary gland, transported into HM across epithelial cells by the polymeric Ig receptor (pIgR) (47). SIgAs may also inactivate viruses (e.g., rotavirus and influenza) within epithelial cells and carry these pathogens and their products back into the lumen, thereby avoiding cytolytic damage to the epithelium. Lysozyme (0.32 g/L in colostrum), another major component in HM, is an enzyme capable of degrading the outer cell wall of Gram-positive bacteria (48).

Cytokines, present in picograms in HM, are small soluble glycoproteins that act as autocrine–paracrine factors by binding to specific cellular receptors, operating in networks and orchestrating immune system development and function (49). They act as messengers to boost the neonatal immune system by communicating with other immune components (50). More particularly in colostrum but also in mature HM, a range of inflammatory cytokines are present in free forms, such as interleukin (IL)-1β, IL-6, IL-8, IL-12, tumor necrosis factor (TNF)-α, and interferon (IFN)-γ, and potentially enhance inflammation (i.e., following bacterial lipopolysaccharides) unlike the immunosuppressive cytokine IL-10, which decreases such inflammatory conditions (51). The primary source of these cytokines is the mammary gland, but leukocytes recovered from HM are capable of secreting them (52). HM also contains an ensemble of growth factors, present at very high concentrations after birth but whose concentrations generally decrease during lactation. Some of these growth factors favor the proliferation and differentiation of epithelial cells and modulate mucosal immune response, such as transforming growth factor (TGF)-β (1–2 μg/L), which is one of the most abundant in HM (53). TGF-β is also an immunosuppressive cytokine involved in the induction and function of regulatory T cells, as well as the regulation of other immune cells such as lymphocytes, macrophages, and dendritic cells, which could induce excessive inflammatory responses to stimuli in the infant gut (53). Colostrum TGF-β is involved in switching IgM to IgA in B lymphocytes of the infant gut mucosa (54).

##### Non-protein Nitrogen

The nitrogen HM content is also composed of non-protein nitrogen (NPN), which represents 5–10% and 20–25% of the total nitrogen in colostrum and mature HM, respectively. It is composed of urea, creatinine, nucleotides, choline and amino alcohols, amino sugars, carnitine, polyamines (see *Metabolites and Bacterial Metabolites* section), N-glycans (see *Carbohydrates* section), free amino acids, and peptides (55). Large individual differences in NPN content in HM are observed, likely because this fraction is composed of a heterogeneous mixture of N-containing substances, such as free amino acids that are known to be influenced by several maternal variables. The origin of many NPN compounds in HM is thought to be the metabolic breakdown products, which filter directly from the maternal plasma and/or derive from normal or pathological metabolism within the mammary gland itself (56). Nucleotides are likely to originate from intact or lysed cells in HM. The exact role of most of the NPN compounds is not yet fully established.

#### Carbohydrates

##### Lactose

Lactose is the major constituent and the main carbohydrate of HM. It represents 30–40% of HM energy content (57). Lactose concentration increases with lactation stage, with the lowest concentration (around 56 g/L) in colostrum to reach an average content of 69 g/L at 120 days postpartum (58). Nevertheless, lactose has the least variable concentration among HM macronutrients throughout lactation.

##### Oligosaccharides

HM oligosaccharides (HMOs) are the third major constituent of HM. The amount of HMOs is generally higher in the early stages of lactation, from 20 to 25 g/L in colostrum to 5–15 g/L in mature milk (59–61).

**Figure 2:**
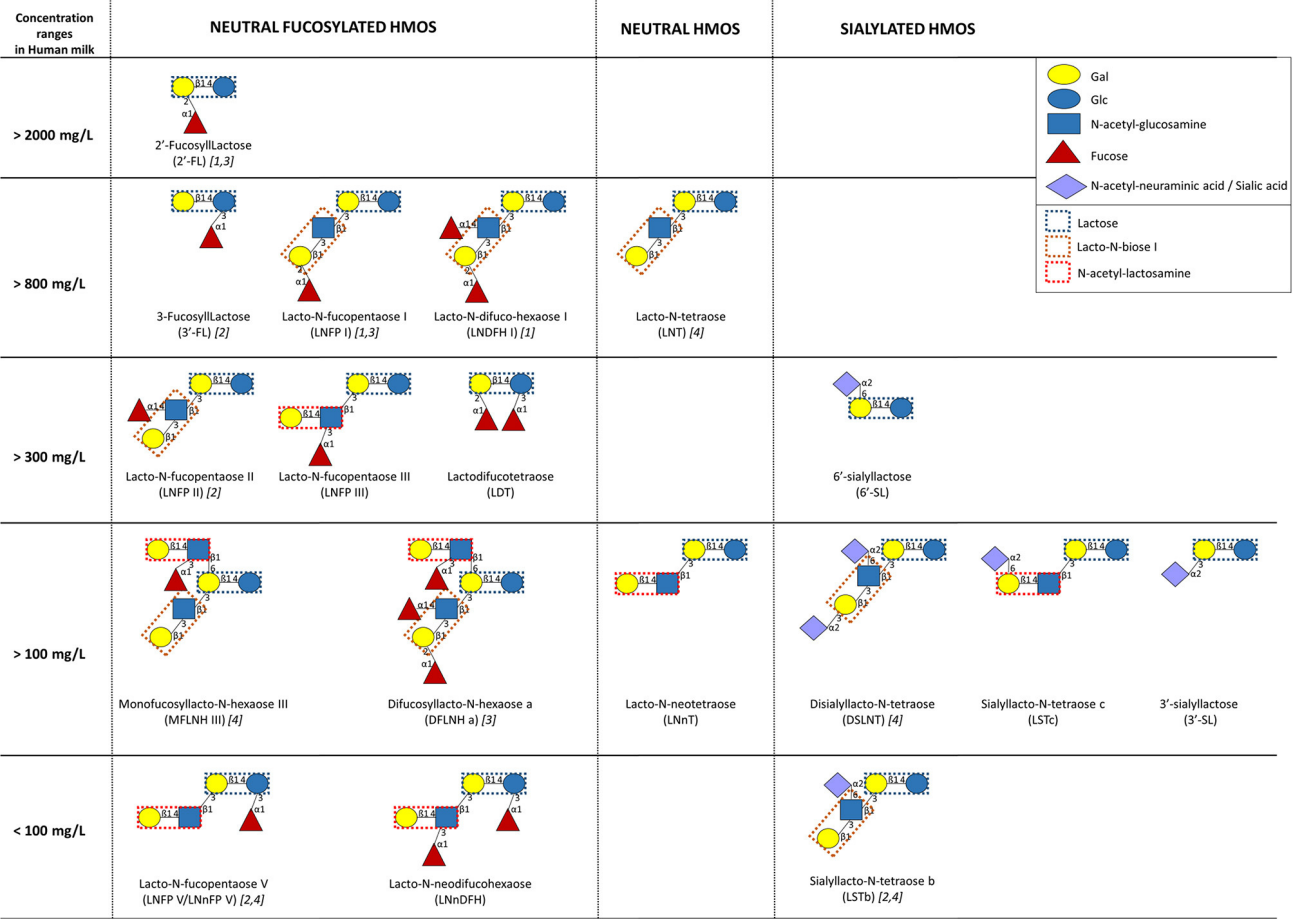
Human milk concentrations of the top human milk oligosaccharides (HMOs) over the first 4 months of lactation (*n* = 290 European healthy mothers), adapted from (67) and (64). [1, 2, 3, and/or 4] indicate the highest HMO concentration in milk of secretor [α1-2-fucosyltransferase FUT2 secretor (Se) gene] and Lewis [α1-3/4-fucosyltransferase FUT3; Lewis (Le) gene] groups (1, Se+Le+; 2, Se–Le+; 3, Se+Le–; 4, Se–Le–).

HMOs are defined as unconjugated molecules with a high level of structural diversity as well as major properties and functions (62, 63). All HMOs contain the disaccharide lactose, branched at the reducing and/or non-reducing ends by a single residue or more, generating more than 100 structurally distinct oligosaccharides. The reducing end glucose (Glc) can be fucosylated in α1-3 linkage, while the non-reducing end galactose (Gal) can be fucosylated in α1-2 linkage, sialylated in α2-3 or α2-6, or even elongated in β1-3 by lacto-*N*-biose I (Galβ1,3-GlcNAc) or in β1-6 by *N*-acetyl-lactosamine (Galβ1,4GlcNAc). Additional branching can occur with fucose, sialic acid (Neu5Ac), and/or *N*-acetyl-lactosamine. Thus, HMOs are named as fucosylated neutral HMOs, non-fucosylated neutral HMOs, and sialylated HMOs. Fucosylated and non-fucosylated neutral HMOs encounter 35–50% and 42–55% of total HMOs, respectively (64). Despite the identification of so far more than 150 structurally different HMOs, the main fraction (~90%) is composed of >20 different ones (65–67) (Figure 2).

Overall, the composition in HMOs in HM depends on genetic and environmental factors. The most important variability in HMO composition remains the genetic capacity of individual women to express α1-2-fucosyltransferase FUT2 (secretor gene, *Se*) and/or α1-3/4-fucosyltransferase FUT3 (Lewis gene, *Le*) in the mammary gland (2, 64). Fucosyltransferase (FUT-2 and/or FUT-3) polymorphisms result in four distinct milk groups: Se+Le+, Se–Le+, Se+Le–, and Se–Le–, which, respectively, represented 72–75, 11–18, 7–11, and 3.5% of European or Brazilian mothers (66, 67). The composition in HMOs in the milk of Se+Le+ mothers presents a higher diversity than the composition of HMOs in the milk of Se–Le– ones. Moreover, non-secretor mothers (Se–Le+ and Se–Le–) secrete a lower amount of HMOs than secretor ones (66, 67). The quantification of 20 HMOs from the milk of 290 European mothers during the first 4 months of lactation showed that 2′-fucosyllactose (FL) and lacto-*N*-fucopentaose (LNFP) I are the most abundant oligosaccharides in milk from secretor mothers (67). On the other hand, the highest oligosaccharides in milk of non-secretor mothers are, respectively, 3′-FL/LNFP II and lacto-*N*-tetraose (LNT)/disialyllacto-*N*-tetraose (DSLNT) (67). However, the genetic mother status (*Se/Le*) does not affect the concentrations of 3′-sialyllactose (SL) and lacto-*N*-neodifucohexaose (LNnDFH) as well as the total neutral core and the total acidic HMOs (66, 67). Despite these general profiles, HMO concentrations present a great variability even in the milk of mothers with the same *Se/Le* status (66). Beside genetic factors, time and mode of delivery also affect the amount and the composition of HMOs. The milk of women with preterm infants is overrepresented by sialylated HMOs, and the concentrations of total HMOs are lower than those of women with term infants (64, 68). Samuel et al. showed that the composition of HMOs is also affected by the mode of delivery at day 2 and day 30 of lactation, specifically with a lower amount of 2′-FL, 3′-SL, and 6′-SL in the milk of women who gave birth through caesarian section (67).

In addition to free oligosaccharides, N-glycans are oligosaccharides attached to the asparagine residues of a protein via *N*-acetylglucosamine linkages. Glycosylation is an important post-translational modification of proteins. More than 70% of HM proteins are highly glycosylated (69). They include LF, lactadherin, SIgAs, mucins, α-lactalbumin, various Igs, and at least 26 other proteins in the whey fraction (70).

### Micronutrients

HM micronutrients include vitamins and minerals. Vitamins provided by HM are all the essential vitamins needed for infant growth. Vitamin composition is linked to maternal nutritional status, specifically liposoluble vitamins like vitamins A and D (10). Furthermore, breastfeeding provides a wide range of trace elements (copper, zinc, barium, iron, cobalt, manganese, cesium, etc.) to the infant. Their concentrations vary throughout lactation and are higher in colostrum than in mature milk, but their concentration is not affected by maternal intake (9, 10).

### Hormones

Many hormones are present in HM, the vast majority being transported into HM from the maternal circulation but several of them being also synthetized within the mammary gland (13, 71). In general, their concentrations in HM are higher than in plasma and in colostrum and transition milk than in mature milk. Their structure may differ from that in plasma due to glycosylation or phosphorylation within the mammary gland before secretion into HM (13). HM hormones include pituitary (prolactin, growth hormone, and thyroid-stimulating hormone), hypothalamus (thyroid-releasing hormone, luteinizing hormone-releasing hormone, somatostatin, gonadotropin-releasing hormone, and growth hormone-releasing hormone), thyroid (thyroxine and triiodothyronine), parathyroid (parathormone, parathormone-related peptide, and calcitonin), steroid (estrogen, progesterone, and adrenal steroids), gut (insulin, ghrelin, and obestatin), and adipocyte (leptin, adiponectin, and resistin) hormones as well as growth factors [epidermal growth factor (EGF), nerve growth factor, insulin-like growth factor (IGF)-I and II, relaxin, and TGF-α and β] (13).

The presence of leptin (72, 73), ghrelin (74), and adiponectin (75) in HM has deserved great interest and has been extensively studied in the last 15 years due to their key role in regulating eating behavior and metabolism (76). Leptin is transferred from the maternal circulation to HM (72), and HM leptin concentration correlates with maternal plasma leptin concentration and maternal body mass index (BMI) (73). Leptin is also produced by mammary epithelial cells and secreted in milk fat globules (77, 78). The production of leptin in breast tissue might be regulated physiologically according to the nutritional state of the infant, as suggested by Dundar et al., who showed different leptin levels in maternal milk of small for gestational age (SGA), large for gestational age, or appropriate for gestational age (AGA) infants (79). Similarly, a remarkable decrease in leptin levels from colostrum to mature milk was also observed in mothers who delivered SGA infants and not in mothers who delivered AGA infants, which may contribute to early catch-up growth of SGA infants (80). Leptin concentrations are also higher in term milk compared with preterm milk (81, 82) even if some contradictory results exist (83). Similar to HM leptin, HM ghrelin comes from maternal plasma (74) and is likely synthesized and secreted from the breast (84). Adiponectin has been measured in skim milk at concentrations higher (more than ×40) than that of the other major adipokines leptin and ghrelin and correlated positively with maternal obesity (74, 75, 85). Adiponectin concentrations were higher in preterm HM compared with term HM (82). HM growth factors (IGF-I, IGF-II, EGF, and insulin) have also been extensively studied due to their gut trophic effects (86). IGF-I and EGF were particularly high in colostrum, while insulin seems to be provided at relatively constant level in colostrum, transitional milk, and mature milk in preterm milk, with their concentrations decreasing postpartum in term milk only, with no difference between term and preterm milk insulin concentrations at delivery (87). In SGA infants, however, a decrease in insulin level from colostrum to mature milk was reported (80). Insulin content in HM is directly in relation with its actual concentration in maternal blood (88).

### Bacteria

Although HM, like other human fluids, has long been considered sterile, microorganisms have emerged as a natural part of HM [for a detailed review on milk microbiota composition and origin, please refer to dedicated reviews (89, 90)]. The first studies focused on the presence of bacteria during intra-mammary infections and the transmission of pathogens through breastfeeding (91, 92). The presence of a complex microbial moiety consisting of commensal bacteria associated with healthy HM is now widely accepted, at least once milk is expressed. Whether a complex and living microbial community can be associated with milk inside the breast and the mammary ducts remains to be determined. The presence of a complex microbial moiety in HM, hereafter referred to as “milk microbiota,” is supported by numerous studies, especially in the last decade, through the use of high-throughput sequencing approaches (89, 90, 93–104) but also culture-dependent analyses (96, 105–107). Some studies considered bacteria isolated from HM as contaminants originating from mother skin and infant oral cavity (11); others suggest that HM bacteria partly originate from maternal gut through a yet-hypothetical entero-mammary pathway. Several questions remain on this complex microbial moiety of HM, in relation to its origin, the factors shaping its composition, its viability, and on its contribution to the establishment of the gut microbiota and subsequent health outcomes in infant.

Characterization of HM microbiota relies on different types of approaches, including culture-dependent and culture-independent approaches such as metataxonomics, based on 16S rRNA gene amplicon sequencing (89). Milk is generally collected after cleaning the breast, by manual expression or using a pump, although some studies also chose to collect milk in a non-aseptic environment and to characterize the “breastfeeding-associated microbiota of HM,” as it is transmitted to infants (103). Methods used to explore HM microbiota are likely to introduce major differences in its composition between studies. Culture-dependent approaches will allow the identification of a fraction of viable bacteria, i.e., those who are cultivable in the growth conditions used, whereas culture-independent approaches will detect DNA of the total bacterial population, independently of their physiological state. Most of the latest approaches rely on amplicon sequencing targeting, mostly the bacterial fraction of microbiota and to lesser extent the fungal community. A few studies based on shotgun metagenomic approaches are now available, giving access to archaeal, fungal, and viral communities and to a prediction of functions of these bacteria (108–110). Besides, within molecular approaches, several technical factors related to sample preparation, sequencing platform, or analytical pipelines may introduce some variability (111–113). Due to the low HM microbial load and the use of PCR-based techniques, these molecular approaches are subject to environmental contaminations during sampling or sample processing, notably by kit reagents, as was established for the “placenta microbiota” (114, 115). The inclusion of negative controls (“reagent only”) is thus important to determine background contamination and ensure subsequent removal of “contaminant reads.” Despite all these sources of variations, the large number of studies has allowed a better characterization and understanding of this complex microbial moiety.

HM microbiota is characterized by a low bacterial load but a high diversity. The total bacterial load was evaluated to be ~10^3^–10^4^ cfu/ml (range 10^1^–10^6^) in healthy HM by numeration on non-selective media, depending on the media used or the collection mode (manual expression *vs*. pump) and ~10^5^–10^6^ cfu/ml by qPCR on total DNA (96, 105, 106, 116). This observation suggests that a part of HM microbiota is either non-viable or non-cultivable. Of note, these bacterial cells were mostly shown to exist in HM in a free-living state and not to be associated with human cells (116). Despite this low bacterial load as compared with the well-characterized gut microbiota, HM was found to harbor a complex and diverse microbiota with several dozens of genera and more than 200 species identified so far (90, 104, 106, 117, 118).

**Figure 3:**
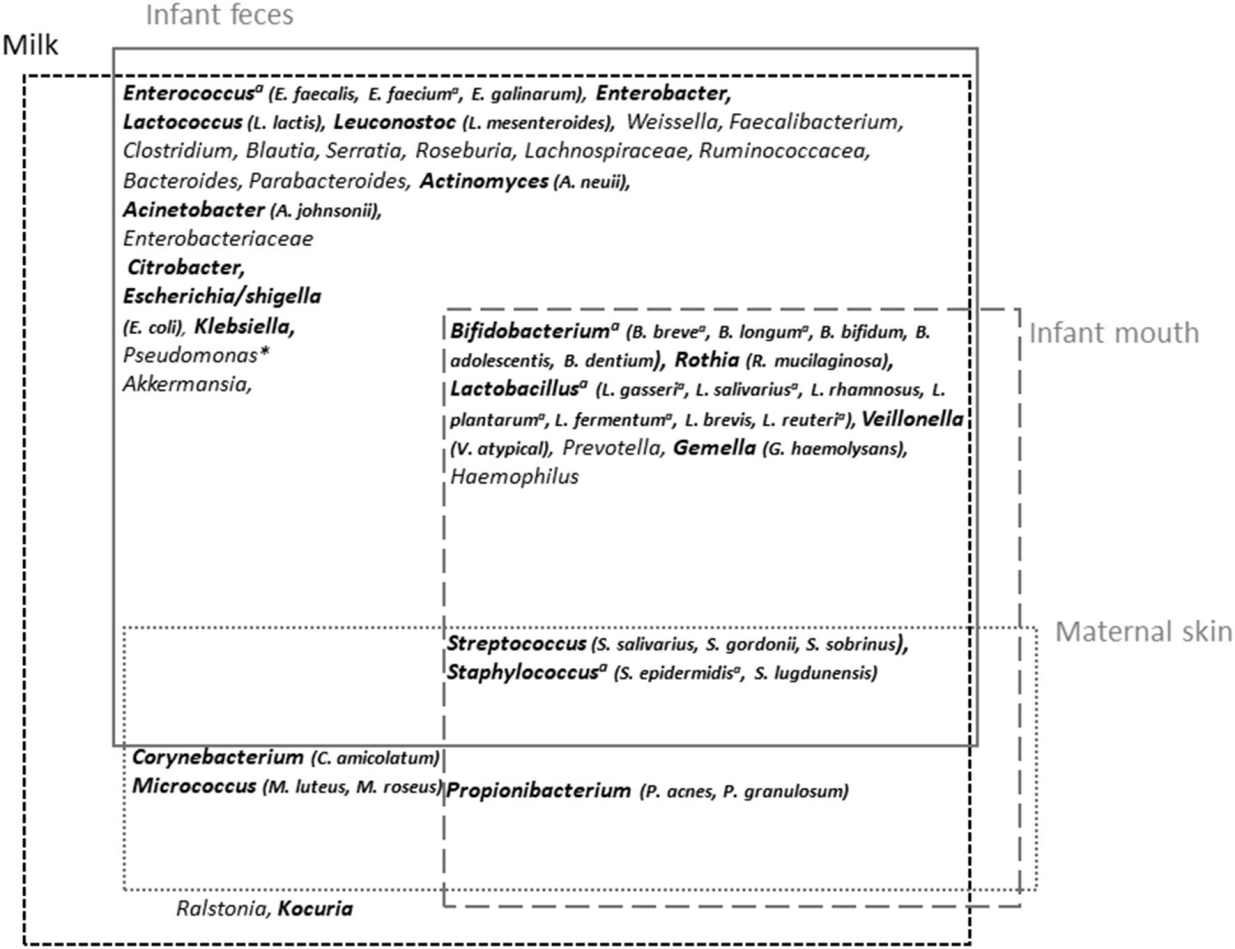
Milk microbiota major taxa and overlap with infant feces and mouth and maternal skin microbiota. Overview of milk microbiota major taxa and their possible origin [based on the reviews by Oikonomou et al. (89) and Jost et al. (90) for milk microbiota and Byrd et al. (119) and Xiao et al. (120) for maternal skin and infant oral microbiota, respectively, and based on comparative studies between microbiota associated with these different sites (98–100, 106, 121)]. The taxa in bold correspond to those for which isolates have been obtained (~viable fraction of milk microbiota). ^a^Taxa for which shared strains between milk and infant feces have been reported; *generally considered as a contaminant.

Among the most frequently cited taxa, *Staphylococcus* and *Streptococcus* have been identified as universally predominant in HM (97). Several additional taxa have been frequently cited, including *Corynebacterium, Bifidobacterium, Propionibacterium, Bacteroides, Enterococcus, Faecalibacterium, Lactobacillus, Veillonella, Serratia, Ralstonia, Acinetobacter, Rothia*, and several members of the Lachnospiraceae and Ruminococcaceae families, suggesting the existence of a core HM microbiota (85, 89, 91, 100, 103) (Figure 3). *Pseudomonas* has also frequently been proposed to be part of HM microbiota, although its presence may be attributed to contamination issues (106).

A cross-species analysis of milk microbiota even suggested that some of these frequently cited taxa could be universally shared within species, thus constituting an inter-species core milk microbiota (89). HM microbiota composition was globally confirmed by culture-dependent studies, albeit with overrepresentation of easily cultivable aerobic or aerotolerant members such as *Staphylococcus, Streptococcus*, and *Propionibacterium* (96, 105, 106). In a study of 31 HM, the combination of cultivation with matrix-assisted laser desorption/ionization time-of-flight (MALDI-TOF) mass spectrometry identification allowed the identification of more than 1,000 colonies (106). In addition to *Staphylococcus* and *Streptococcus* isolates, which were dominant in all HM samples, other highly abundant genera, present in >50% of the samples, belonged to *Acinetobacter, Gemella, Rothia, Corynebacterium, Veillonella, Lactobacillus, Enhydrobacter*, and *Propionibacterium* (Figure 3). Isolation of obligate anaerobic species such as strict anaerobic *Bacteroidetes* or *Clostridium* members was improved following milk storage for 6 days at 4°C, suggesting that these taxa also belong to the viable fraction of milk microbiota despite poor retrieval up to now (105). The fungal and viral HM communities have also started to be explored (122–124). The presence of fungi including *Saccharomyces* species has been reported in HM samples (122).

The origin of HM microbiota remains a matter of debate. HM microbiota is likely a combination of microorganisms originating from maternal skin or even mammary tissue (125) and infant oral cavity. It may also result from the maternal digestive tract through a yet-hypothetical entero-mammary pathway involving immune cells (90). In agreement with the two former sources, *Propionibacterium* sp., *Staphylococcus* sp., or *Corynebacterium* sp. are usual members of the adult skin microbiota, and the presence of several members of the infant oral cavity in HM has been reported (104, 118). The infant oral microbiota contribution to HM microbiota was evaluated to be ~21 and 66% 2 days and 5 months after birth, respectively (101). In agreement with the inoculation of HM microbiota by the infant mouth through a retrograde flow back into the mammary duct during suckling, Biagi et al. reported enrichment of HM microbiota by typical oral bacteria such as *Streptococcus* and *Rothia*, after the infant latching to the mother's breast, compared with HM microbiota collected by pump (99). Kordy et al. (126) also reported maternal areolar skin and infant oral cavity as major source of the breast milk microbiota, with an average contribution of 46 and 26%, respectively. Albeit controversial, the existence of an entero-mammary pathway has been proposed (127). This route is supported by a partial overlap between maternal feces and HM microbiota compositions (101, 128). In a study comparing the milk, vaginal, and fecal microbiota, Avershina reported a low redundancy in terms of bacterial species between these three microbiota, but HM had higher intra- than inter-individual similarities toward both vaginal and stool samples, supporting, to a certain extent, the translocation of gut microbiota to the milk (128). Using shotgun metagenomic sequencing, Kordi et al. identified the same strain of *Bifidobacterium breve* in maternal rectum, breast milk, and the stool of an infant delivered via caesarian section, suggesting direct transmission from maternal gut (126). The existence of the endogenous route is also supported by the isolation of common strains of *Bifidobacterium longum* from maternal and neonatal feces as well as from HM (46). Additionally, oral administration of some lactobacilli strains to lactating women led to their presence in milk (129, 130). This entero-mammary pathway may account for the presence of DNA corresponding to major gut-associated obligate anaerobes, including *Bacteroides, Prevotella, Blautia, Clostridium, Dorea, Eubacterium, Coprococcus, Faecalibacterium*, or *Roseburia* (90).

Several factors have been proposed to shape HM microbiota composition. Strong inter-individual variations may be the result of both mother-related and environmental factors (89, 102, 104, 131). HM microbiota changes with time and notably between colostrum and mature milk (104, 116, 118). Differences were also reported in relation to the delivery mode, BMI, and parity (102, 104, 132–134). However, depending on the study design or methods used, contradictory results reporting the lack of effect of most of these factors have also been proposed, including the lactation stage (102), the delivery mode (caesarian section vs. vaginal delivery), the gestation length (preterm *vs*. term) (135), or the mother's BMI (136). The mode of breastfeeding itself was shown to affect HM microbiota. Pumped HM was associated with a higher abundance of potential pathogens and lower abundance of bifidobacteria and oral cavity-related species (99, 106, 134). Finally, HM microbiota is clearly affected by the mother health status including mastitis development and antibiotherapy or chemotherapy, which can directly affect microbial diversities and profiles (89, 108, 137–139).

### Metabolites and Bacterial Metabolites

HM also contains many small molecules (<1,500 Da), which have recently deserved much interest due to their potential role on infant growth as well as on the development of the gut, immune, and nervous systems and other tissues (140). These small molecules include molecules found in milk fat globules (triacylglycerol species, glycerophospholipid species, sphingomyelin species, cholesterol, etc.) as well as proteins and peptides (proteinogenic amino acids), disaccharides and oligosaccharides (glucose, galactose, fucose, etc.), and other components dissolved in HM (amino acids, creatinine, urea, citrate, 2-keto-glutarate, choline, nucleotides, polyamines, etc.) (140). They have been identified using metabolomics methodologies (nuclear magnetic resonance spectroscopy or mass spectrometry); and depending on the technique and HM sample preparation, from hundreds (141, 142) to more commonly dozens of metabolites have been described in HM (143, 144). HM metabolite concentrations change with the duration of lactation (143), notably carbohydrates and amino acids during the first month of lactation (140), with high levels of amino acids in colostrum and high levels of saturated acids and unsaturated acids in mature milk (145). There are also differences in preterm and term HM metabolite concentrations, mainly in early lactation (140, 146). HM metabolites differ across specific geographical locations (China *vs*. Finland vs. South Africa, for instance) (144, 147). The pathophysiologic status of the mother influences the metabolite content of HM. HM of women with irritable bowel syndrome displays less sugar metabolites (lactose) and 2-aminobutyrate and more energy metabolites (succinate and lactate) than HM of healthy mothers (148). Likewise, gestational diabetes mellitus is associated with alterations in the metabolome of HM, especially the colostrum (145).

HM metabolites may be filtered from the mother bloodstream through the mammary epithelium, may originate from different metabolic processes within the mammary gland, or may be produced through the metabolic processes of resident microbes in HM (149). It is difficult to ascertain the microbial origin of HM metabolites, as many metabolites can be produced by both bacteria and eukaryote cells, but some HM metabolites are more likely to be of bacterial origin. This is the case of biogenic amines including the polyamines (spermine, spermidine, and putrescine), together with the monoamines (tyramine) and diamines (histamine and cadaverine) (149). *Enterococcus*, a major bacteria group in HM, are the main producers of biogenic amines, mainly putrescine and tyramine (150). A positive correlation between putrescine concentration and *Pseudomonas fragi*, a Gammaproteobacteria, has recently been described in HM (151). HMOs could also be a direct substrate for HM bacteria, which would produce metabolites. However, to our knowledge, the correlation between specific products of HMO fermentation and HM bacterial strains has never been described. In a recent study, Mai et al. demonstrated *in vitro* that HM promoted the growth of probiotic *Lactobacillus reuteri* DSM 17938, a strain originally isolated from HM, and its secretion of potentially beneficial metabolites (such as succinate, glutamine, *N*-acetylcysteine, citrulline, spermidine, and lactate) (152), suggesting that HM could indeed favor the growth and metabolism of HM bacteria, generating specific bacterial metabolites.

## Role of the Different Breast Milk Components in Shaping the Infant Gut Microbiota

### Oligosaccharides

Among HM components, HMOs, which are both non-digestible molecules utilized by commensal infant bacteria in the large intestine and free competitor to enteric pathogens, are known to strongly influence the composition of the infant gut microbiota. Several studies have shown that the fecal bacterial composition of breastfed infants is different from that of formula-fed infants (64, 153). The fecal microbiota composition of formula-fed infants devoid of HMOs is poorer in bifidobacteria than that of breastfed infants. While the microbiota of breastfed infants was represented by 90% of bifidobacteria and lactobacilli, that of formula-fed infants was composed of 40–60% bifidobacteria and lactobacilli, and the remaining represented by Enterobacteriaceae and *Bacteroides*. In addition, the rate of establishment of gut microbiota in infants breastfed by secretor mothers is faster than in those breastfed by non-secretor mothers (64, 154). Furthermore, the microbiota composition of breastfed infants from non-secretor mothers was shown to be slightly different from the microbiota of infants breastfed by secretor mothers with higher colonization by *Bifidobacterium adolescentis* and absence of *Bifidobacterium catenulatum* (155). These latter observations demonstrated the major role of HMOs in the establishment of the infant gut microbiota.

The predominant members of the early gut microbiota, *Bifidobacterium, Bacteroides* spp., and *Lactobacilli*, possess the ability to utilize HMOs by fermentation, while other members, including *Clostridium, Enterococcus, Escherichia, Eubacterium, Staphylococcus, Streptococcus*, and *Veillonella* spp., do not (64, 90, 156–159). *In vitro* analyses showed that the major *Bifidobacterium* strains (*Bifidobacterium breve and Bifidobacterium bifidum*) present in the infant gut microbiota were also the major strains able to ferment HMOs (159). Moreover, Borewicz et al. showed a relation between HMO consumption patterns and specific microbial groups affecting both bacteria possessing the ability to utilize HMOs and the others (160).

The prebiotic role of HMOs on the infant microbiota can be partly attributed to their specific structures. HMO consumption is mainly associated with *Bifidobacterium* genus but is also found in a few *Bacteroides* and *Lactobacillus* species. However, the ability to consume HMOs is not characteristic of all bifidobacterial isolates, and certain HMOs are more utilized by bifidobacteria than others (161). Moreover, cross-feeding between HMO degraders and non-HMO users has been observed (162). Genomics, transcriptomics, and glycobiology methods have been useful to study the molecular basis of this preferential utilization of HMOs by bifidobacteria species, especially the induction of specific genes in the presence of HMOs, which confer a selective advantage on this substrate (161). As a consequence of this preferential use of HMOs by some specific strains, analyses of the HMOs and the fecal microbiota composition of 1- and 3-month-old breastfed infants showed that 2′-FL and LNFP-I, which are the main oligosaccharides found in the milk of secretor mothers, affect the infant gut microbiota (160, 163). Among the synthetized HMOs, 2′-FL and lacto-*N*-neotetraose (LNnT) are widely studied and are considered safe for infant nutrition. Fecal microbiota composition of 2′-FL- and LNnT-supplemented formula-fed infants was more similar to that of breastfed infants, in terms of microbial diversity, global composition at the genus level, and abundance of several major genera than that of infants fed a non-supplemented formula (64, 164, 165). Moreover, 2′-FL and LNnT supplementation was associated with lower prescription of antibiotics during the first year of life, although fecal microbiota profiles no longer differed between supplemented and non-supplemented infants at 12 months of age (165). Likewise, sialic acid is known to be an essential nutrient during periods of rapid neural growth and brain development in the newborn (166). α2-6-Linked sialylated oligosaccharides were present in greater proportion than the α2-3-linked structures during early lactation (167, 168). Recently, Bondue et al. demonstrated the ability of a specific Bifidobacteria, *Bifidobacterium mongoliense*, to utilize 3′-SL as the main source of carbon (169).

Individual or mixed HMOs also have a preventive role in the attachment of pathogens in the infant gut. Some HMOs mimic lectins or glycan-binding proteins, preventing pathogen attachment on epithelial surfaces. 2′-FL was reported to alleviate inflammation, lower allergic reaction, and prevent enteric pathogens (such as *Campylobacter jejuni* or *Escherichia coli*) attachment on epithelial surfaces (170, 171). α1-2-Fucosylated HMOs act as antiadhesive antimicrobials against *C. jejuni* (170, 171). For some pathogens such as *Entamoeba histolytica*, complex HMOs containing Gal/GlcNAc patterns (LNFP II and LNFP III but not LNFP I, which contain α1-2-fucose residue) are required to block attachment or cytotoxicity (172). Interestingly, 2′-FL and 6′-SL were found to directly bind to TLR4 and inhibit TLR4 signaling in *ex vivo* gut tissue and organoid cultures, explaining the protection against the necrotizing enterocolitis in newborn mice and premature piglets (173). Recently, Wang and collaborators demonstrated in mice that 2′-FL intake increased the abundance of *Akkermansia* spp., a probiotic potentially involved in the expression of mucins in goblet cells and thus the reduction of the colonization of the harmful bacteria *E. coli* O157 (174).

### Milk Bacteria

Considering an estimated daily ingestion of log 5 to 7 HM-associated bacteria, HM microbiota is a continuous source of commensal or probiotic microbes able to colonize the gut or influence the infant gut microbiota during the first stage of life (90, 101, 118). Strong overlap exists between milk and the infant gut microbiota when considering major taxa of milk microbiota (Figure 3). Several studies intended to evaluate the role of HM microbiota in the infant gut colonization, revealing some discrepancies between them due to both the methods and the taxonomic levels used to compare microbiota. A strong overlap between the infant gut and milk microbiota was pointed out by Pärnänen et al. in a metagenomic analysis, as 76% of the species found in milk were present in the infant gut (110). In this study, a strong overlap was also revealed for antibiotic resistance genes (ARGs) and mobile genetic elements (MGEs) between milk and infant feces, as 70% of the ARGs detected in milk were present in infant feces. Conversely, infant feces shared 20% of their ARGs and 12% of their MGEs with HM. William et al. estimated a direct contribution of only 4.9% of HM microbiota to the infant gut microbiota and suggested indirect contribution through an effect on microbiota in the upper part of digestive tract, including the oral microbiota (101). Despite this low direct contribution of HM microbiota to the infant gut microbiota, these two communities were found to be intimately linked as revealed by correlation analyses (101). Using a similar tool (i.e., SourceTracker), Pannaraj et al. estimated the proportion of bacteria in infant stool originating from HM to be 27.7 and 10.4% for primarily breastfed and non-primarily breastfed infants in the first month of life, and this contribution decreased thereafter (175). In agreement with the role of HM microbiota in shaping the infant gut microbiota, the infant gut microbiota and even the resistome were more similar to each infant own mother's gut microbiota than to unrelated women (99, 110). Likewise, Biagi et al. (99) investigated the relation between HM, oral microbiota, and fecal microbiota in preterm infants whose breastfeeding mode changed from indirect intake through breast pump to direct breastfeeding. A non-supervised approach allowed defining three HM bacteria community types that were more or less related to the breastfeeding mode. Interestingly, compositional differences between these milk community types were associated with compositional differences in infant fecal and oral microbiota. Similar conclusions were supported by a study based on nearly 400 mother–infant dyads in 11 international sites (100). In this study, despite limited associations between individual genera in HM and fecal microbiota, community-level analyses suggested strong, positive associations between these two microbiota. Similar conclusions were drawn regarding the viral communities, which were distinguishable between HM and infant feces, but with a significant number of shared viruses in HM and feces from all mother–infant dyads (109). Thus, although all these studies differ in their rates of overlap and contribution, depending on the methods used, the cohorts, and whether the reference is HM or infant feces, they support partial overlap between HM and infant gut microbiota and suggest that they are both positively linked.

Shared species between HM and infant gut include the pioneer genera initiating gut microbiota assembly (46). They include facultative anaerobes such as *Staphylococcus, Streptococcus, Lactobacillus, Propionibacterium, Enterococcus*, or *Escherichia* species that contribute to generate an anaerobic environment and favor the subsequent implantation of obligate anaerobes such as *Bifidobacterium, Bacteroides, Blautia*, or *Veillonella* species (90, 176). Several studies reported the vertical transfer of *Bifidobacterium* species, which are dominant in breastfed infant gut (96, 124, 177). Biagi et al. (98) characterized the composition of the oral and fecal microbiota of infant and that of HM microbiota in 36 healthy mother–infant pairs and reported a limited number of operational taxonomic units (OTUs) shared among the three microbiota that belonged to the *Bifidobacterium* genus, as well as specific *Streptococcus* and *Staphylococcus* OTUs. These *Streptococcus* and *Staphylococcus* OTUs were dominant in the infant mouth ecosystem as well, supporting the baby's mouth as a transition point between HM and infant gut, contributing to both infant gut and mother's milk duct colonization.

The use of 16S rRNA gene-based molecular approach to investigate vertical transfer may be limited and subject to criticisms. Others studies combining culture-dependent approaches with genotyping of isolates reported the presence of the same strains in infant feces and HM, supporting a vertical transfer of both facultative and strict anaerobes (46, 90, 124, 178). In particular, few studies reported the presence of shared strains of *B. breve* and *Bifidobacterium longum* in HM and infant feces (46, 107, 177). Exploration of the *Bifidobacterium* and bifidophage population in the maternal and infant feces and HM of 25 mother–infant pairs through the combination of molecular and culture-dependent approaches revealed that similar OTUs or strains as well as bifidophages were shared between these three types of samples within mother–infant pairs (124). Apart from *Bifidobacterium* species, the presence of shared strains belonging to *Staphylococcus, Enterococcus*, and *Lactobacillus* in HM and infant feces was also demonstrated through genotyping of isolates (Figure 3) (90, 107, 178–180). Martin et al. (177) notably reported the presence of two to four shared strains of *Staphylococcus, Lactobacillus*, and/or *Bifidobacterium* between HM and infant feces from 19 mother–infant pairs (177). Transfer of other strict anaerobes such as *Bacteroides* or *Veillonella* species still remains to be clearly demonstrated by culture-dependent methods. Additional studies based on high throughput culturomic approaches may help to evaluate the proportion of shared strains between HM and the infant gut microbiota.

An alternative to identify HM bacteria that are able to colonize the gut was proposed by Wang et al. (181). By inoculating normal chow-fed germ-free mice with HM, they reported the presence in the feces of OTUs belonging to *Streptococcus, Staphylococcus, Corynebacterium*, and *Propionibacterium* genera as well as anaerobic gut-associated bacteria belonging to *Faecalibacterium, Prevotella, Roseburia, Ruminococcus*, and *Bacteroides* at low abundance. *Bifidobacterium* was also isolated from mice feces at very low abundance, although it was below the detection limit in HM (181). Of note, although some species were shared between HM and infant feces, their relative abundance within microbiota strongly differs. This is the case for *Bifidobacterium* whose abundance was low in HM but which became dominant in the infant gut, due to modifications of growth conditions and to their ability to metabolize HMOs (4, 90).

Beyond a direct role in seeding the infant microbiota, HM microbes likely contribute to gut microbiota assembly through their effects on gut microbes, including competition for nutrients or gut mucosal binding sites, direct inhibition, or contribution to trophic chains. Hence Jost et al. (90) suggested a role of HM bacteria in gut lactate metabolism. Most of HM bacteria are involved in either lactate production (*Staphylococcus, Streptococcus*, and *Lactobacillus*) or lactate utilization (*Propionibacterium* and *Veillonella*), which could favor the establishment of balanced metabolic activities in the gut and prevent disorders related to lactate accumulation but also influence gut microbiota establishment through this trophic chain. Regarding inhibition potential of HM microbiota, HM contains several bacteriocin-producing strains, such as *Enterococcus faecalis, Enterococcus faecium*, and *Staphylococcus* sp., that may provide them a competitive advantage in the colonization of the infant gut or contribute to shaping of the infant gut microbiota (182). Likewise, HM contains bacteriophages that are partly transmitted to the infant gut and that could influence the infant gut microbiota composition (109). Finally, HM microbiota may also influence the infant gut microbiota assembly through their effect on gut immune system via their immunomodulation properties (modulation of cytokine production and induction of SIgAs) or their impact on gut barrier function (183).

As previously mentioned, part of the HM microbiota is non-viable or, at least, non-cultivable. This was revealed by discrepancies between the total bacterial load of HM as determined by culture-dependent or metataxonomic approaches and differences in the microbial profile with overrepresentation of few genera in culture-dependent approaches (96, 105). However, strains corresponding to obligate anaerobes have been isolated (105), suggesting that the living part of HM microbiota is underestimated. Further exploration of HM microbiota using high-throughput culture-dependent methods is now required to fully understand the contribution of HM microbiota to the infant gut microbiota. Moreover, even if the living part of HM microbiota is underestimated, part of this microbiota is likely inactivated during the first steps of digestion. This raises questions about the role of this “non-living” part of the microbiota, since bacterial antigens would still be able to interact with the host immune system and indirectly contribute to the shaping of infant gut microbiota.

### Immune Factors

#### Lactoferrin

LF has a direct cytotoxic effect against a large panel of microorganisms (bacteria, viruses, and fungi), mainly in the gut mucosa. For example, the iron-free form of LF can kill *Streptococcus mutans, Streptococcus pneumoniae, E. coli, Vibrio cholera, Pseudomonas aeruginosa*, and the fungal pathogen *Candida albicans* (184). Moreover, LF also has bacteriostatic properties due, in part, to its ability to bind ferric ions and most of the iron from HM, thus reducing iron availability for bacteria. Multiple clinical studies have suggested a number of potentially favorable biologic effects associated with LF in infants and children. The first randomized controlled trial assessing LF supplementation in neonates reported a reduction in the incidence of late-onset sepsis in bovine LF supplemented compared with placebo in preterm infants (185). Data from the recently completed ELFIN (enteral LF in neonates; *N* = 2,200) and LIFT (LF infant feeding trial; *N* = 1,500) studies will help clarify the potential benefits of LF supplementation in preterm infants (186). Moreover, fragments of human LF and of pIgR stimulate the growth of a large set of *Bifidobacterium* strains. Indeed, the fragments of LF and pIgR are 100 times more effective to enhance *Bifidobacterium* growth on a molar basis than the carbohydrate *N*-acetyl glucosamine, a currently known bifidogenic carbohydrate, leading to the assumption that the bifidogenic activity of HM based on peptides exceeds that of some HM carbohydrates (187).

#### Secretory IgA

In suckling infants, SIgAs shape the composition of the gut microbiota. Immune exclusion is one of the most commonly proposed mechanisms by which SIgAs block microbes from attaching to, colonizing, and invading mucosal epithelial cells. Indeed, SIgAs in HM inhibit the binding of *Clostridium difficile* toxin A to enterocyte brush border membrane receptors (188). Moreover, secretory component (SC) alone is sufficient to inhibit toxin binding to receptors. SC is primarily responsible for blocking toxin A attachment to epithelial cell monolayers. Furthermore, SC may serve as a decoy receptor for other pathogens, including entero-toxigenic *E. coli* (189).

#### IgG

The presence of IgG in HM helps in counteracting the infant deficiencies in opsonization and antibody-mediated cytotoxicity. Antibodies that recognize antigens expressed by entero-toxigenic *E. coli* and other Enterobacteriaceae species of the maternal microbiota are produced and secreted in HM (190). IgG is also important for establishing homeostasis with regard to the newly colonizing microbiota by prevention of the activation of the gut-associated lymphoid tissue (191).

#### Lysozyme

Lysozyme, also called *N*-acetyl muramidase, hydrolyses peptidoglycan polymers of bacterial cell walls at the β1-4 bonds between *N*-acetyl muramic acid and *N*-acetyl glucosamine, thereby lysing Gram-positive bacteria. *In vitro* study using electron microscopy demonstrated that lysozyme can act synergistically with LF to help in bacterial clearance (192). LF first binds to the lipopolysaccharides of the outer cell membrane of the Gram-negative bacteria, creating holes in the membrane. Lysozyme can then enter and degrade the peptidoglycan of the bacteria, killing the pathogens (192).

#### Cytokines

Cytokines participate in the establishment and maintenance of tolerance to harmless food antigens and commensal bacteria (53). However, their precise role in shaping the infant gut microbiota still needs to be demonstrated.

Although many data obtained *in vitro* indicate a possible effect of HM immune factors in modulating the infant gut microbiota, this effect is not supported yet by clinical or animal model studies, except for LF. Further studies are therefore needed to fully assess their role.

### Bacterial Metabolites

It is difficult to speculate on the role of HM bacterial metabolites, as, as seen earlier, the specific bacterial origin of HM metabolites is still difficult to ascertain. Polyamines are mainly bacterial end products and do not interfere with bacterial growth. Thus, HM polyamine content is unlikely to modulate the infant gut microbiota. If lactate, short-chain fatty acids, and intermediary metabolites such as succinate, are effectively produced by HM bacteria and released in HM, then a differential production of these metabolites could interfere with infant microbiota, but such a direct link is speculative, and further studies on the specific role of HM bacterial metabolites on shaping of the infant gut microbiota are warranted.

### Macronutrients

#### Lipids

The role of HM lipid fraction on the infant gut microbiota is poorly documented, but several lines of evidence point to a possible effect. Indeed, *in vitro* studies reported either bactericidal activities of milk lipids, including medium-chain fatty acids (MCFAs), sphingosine, and monoacylglycerols (193), or a bacterial growth promotion activity, especially a beneficial effect of oleic acid on *Lactobacillus* species (194). Accordingly, Nejrup et al. observed significant changes in infant fecal microbial communities (increased *Lactobacillus* and *Bifidobacterium* abundances and decreased Enterobacteriaceae abundance) cultured with selected HM lipids MCFA, monoacylglycerol, and/or sphingosine during anaerobic *in vitro* fermentation (195). Investigations of the effect of lipid fractions of infant formulas on the infant gut microbiota or of associations between HM lipid fractions and infant microbiota composition are also available. Increasing the proportion of palmitic acid in the sn-2 position of triglycerides in infant formula increased fecal *Lactobacillus* and *Bifidobacterium* counts after 6 weeks (196). A significant association between the proportion of decanoic acid, myristic acid, stearic acid, palmitic acid, arachidonic acid, and docosahexaenoic acid in the sn-2 position of triglycerides in HM of Chinese women and *Bacteroides*, Enterobacteriaceae, *Veillonella, Streptococcus*, and *Clostridium* abundance of their infant gut microbiota has recently been described (197). HM gangliosides could also participate in the shaping of the infant gut microbiota. They are glycosphingolipids consisting of a hydrophobic ceramide and a hydrophilic oligosaccharide chain and have been described as putative decoys that interfere with pathogenic binding. Infant formula enriched in ganglioside reduced *E. coli* counts and slightly increased bifidobacteria counts (+0.5 log/g feces) in preterm infant feces after 30 days (198). Likewise, the HM sphingolipids could affect the gut microbiota since several reports in mice indicated an effect of dietary bovine sphingolipids on microbiota composition (199). Yet their effect on the infant gut microbiota has not been investigated to our knowledge. Finally, addition of milk-fat globule membranes to formulas in neonatal piglets shifted their fecal microbiota toward the composition of sow-reared piglets as opposed to plant lipid-based formula (24), with similar results in rats (200).

#### Carbohydrates

##### Lactose

Beside HMOs, whose role in shaping the infant gut microbiota has been discussed above, HM lactose could also contribute, yet to a lesser extent, to the infant gut microbiota establishment. Although large amounts of lactose are unlikely to reach the large intestine due to its hydrolysis and absorption within the small intestine, lactose is easily degraded by several bacterial species (201–203). An association between lactose concentration and the colonic microbiota composition in formula-fed piglets has been described (204). *In vitro* data also suggest a synergy between lactose and oligosaccharides on *B. longum* growth (205). Similarly to lipids, the data on the role of lactose on the infant microbiota are scarce. Consumption of a lactose-reduced and added-sugar (corn-syrup solids) formula by infants for 6 months slightly increased the diversity (+18%) and Acidaminococcaceae abundance (+0.7 log/mg feces) in feces compared with lactose-containing formula consumption (206). These effects were not reproduced in a preterm piglet model where diversity was lower in corn-syrup solid formula-fed piglets compared with lactose formula-fed ones (207).

##### N-Glycans

The role of N-glycans in shaping the infant gut microbiota has been highlighted in a piglet study evaluating the postnatal concentration of N-glycans in sow milk and the piglet microbiota composition in parallel. This study indicated that milk N-glycome correlated to abundances of certain gut microbes, either positively or negatively (208). However, data on correlations between human infant gut microbiota and HM N-glycans are not available yet to our knowledge. The enzymatic equipment and catabolic pathways to use these N-glycans have been identified in certain isolates of commensal *Bifidobacterium* (209) and *Lactobacillus* (210). Some infant-borne bifidobacteria such as *B. longum* subsp. *infantis* were found to harbor a cell-wall associated endo-β-*N*-acetylglucosaminidase able to release oligosaccharides from milk proteins (209). These milk glycoprotein-derived oligosaccharides can serve as selective substrates for the growth of these infant-associated bifidobacteria, similar to HMO (211). However, to our knowledge, data on correlations between human infant gut microbiota composition and HM N-glycans are not available.

#### Proteins and Non-protein Nitrogen

The impact of HM proteins and NPN compounds on the infant gut microbiota has been suggested for proteins and peptides with immunomodulatory properties (described above). Yet other HM proteins and NPN compounds could also affect the infant gut microbiota composition. Several animal studies evaluated the impact of whey protein content in formula on gut microbiota composition. Colonic microbiota diversity and relative abundances of Clostridiaceae, Enterobacteriaceae, *Streptococcus*, and *Streptomyces* were increased in preterm piglets receiving a formula with α-lactalbumin-enriched whey protein concentrate for 19 days (212). However, this was not reproduced in term infants since a formula enriched in α-lactalbumin and glycomacropeptides did not affect fecal microbiota composition in 6-month-old term infants (213). Likewise, a whey or whey-and-casein formula did not affect the gut microbiota in preterm piglets (214). HM mucins may also affect the gut microbiota implantation or at least protect from pathogens. Indeed, mucins have been shown to inhibit some pathogens like rotavirus by inhibiting its replication (215, 216) or *Salmonella enterica* serovar Typhimurium by inhibiting its binding properties on host cells (217).

Although whey protein and casein do not seem to be major HM components in orientating gut microbiota composition, other data support a role of NPN compounds. Indeed, recent work by the Sela group indicated that several *B. infantis* strains were competent for urea nitrogen utilization and that urease gene expression and downstream nitrogen metabolism pathways were induced during NPN utilization (218). Nucleotides may also drive the gut microbiota development since a nucleotide-enriched formula was shown to reduce the *Bacteroides*–*Porphyromonas*–*Prevotella* group to *Bifidobacterium* species ratio in the feces of 20-week-old healthy infants, compared with standard formula (219).

### Hormones

HM hormones retain their biological activity in the infant gut, possibly due to post-transcriptional modification in the mammary gland before secretion into HM, which may increase their resistance to digestion (13). If their role in favoring proliferation of intestinal cells, increasing mucosal growth, enterocyte migration rates, villus height, brush border enzymes activity, and expression of glucose transporters (220) as well as their effects on metabolism through their absorption in infant plasma (76) is well documented, their role as contributors to the colonization patterns of the infant gut microbiome is much less documented. A recent study by Lemas et al. in 2-week-old exclusively breastfed infants highlighted a positive association between HM insulin and both microbial taxonomic diversity and Gammaproteobacteria abundance (e.g., Enterobacteriaceae), whereas HM insulin was negatively associated with Lactobacillales abundance (e.g., Streptococcaceae) (221). As suggested by the authors, this may be due to a direct role of insulin to regulate enterocyte maturation and/or the ability of oral insulin to increase glucose concentration in the gut lumen. As Enterobacteriaceae are a family of glucose metabolizers, their gut colonization could therefore be favored.

In the same study, metagenomic analysis showed that HM leptin and insulin were associated with decreased bacterial proteases implicated in gut permeability and reduced concentration of pyruvate kinase, a biomarker of pediatric gut inflammation (221). There was no association between HM leptin and microbiota (221) even if a role of leptin in modulating gut microbial composition has been suggested in rodent; but this effect, mediated by differential expression of the mRNA expression of gut antimicrobial peptides, did not imply gut leptin receptors (222). However, it has recently been evidenced in rats that supplementation during the first 21 days of life with leptin or adiponectin decreased the abundance of the Proteobacteria phylum and the presence of *Blautia* (223). Moreover, leptin-supplemented rats had lower relative abundance of *Sutterella* and a higher proportion of *Clostridium* genus, among others. Supplementation with adiponectin resulted in lower abundance of the *Roseburia* genus and a higher proportion of the *Enterococcus* genus (223). Oral insulin may also have an antimicrobial action against potential pathogens through upregulation of a specific endotoxin receptor on the gut brush border membrane, as demonstrated in suckling mice receiving insulin orally every day (224).

## Impact of Maternal Diet on Milk Composition: A Nutritional Strategy to Shape the Infant Gut Microbiota Assembly

The impact of maternal diet upon HM macronutrient, micronutrients, and immune factors has been reviewed recently (20). But it is not presented here, so to concentrate on the impact of maternal diet on HMOs, bacteria, hormones, and bacterial metabolites, which are less documented.

### Human Milk Oligosaccharides

Besides genetic factors presented above, physiological and environmental factors such as maternal nutritional status, geographical origin, or type of delivery were shown to affect the composition and amount of HMOs. However, only few studies investigated the impact of maternal diet on HMO composition. In extreme environmental conditions, maternal nutritional status, due to seasonal fluctuations that affect food reserves and diversity in Gambia, was shown to affect the amount of HMOs. Gambian mothers (*n* = 12) who gave birth during the wet season where food is highly depleted had a lower amount of HMOs than mothers who gave birth during the dry season (*n* = 21) (61). An effect of high protein or fiber content in maternal diet during pregnancy and lactation was also suggested in a rat study, with an increase of a neutral oligosaccharide and an acidic oligosaccharide among identified oligosaccharides (225). A recent study observed the presence of the diet-derived sialic acid Neu5Gc in HMOs in 16 samples of HM (226). Because of the human inability to synthesize Neu5Gc, its presence in HM is a clear evidence of a direct influence of maternal diet on HMO biosynthesis, although the positive association observed between ingested and observed (in HMOs) Neu5Gc levels was not significant. In addition, total fruit intake and cured meat intake, positively and negatively, respectively, correlated with the abundance of several HMOs, while cheese intake positively correlated with Neu5Gc levels (226). Moreover, a preliminary study showed that lower BMI (14–18 compared with 24–28) correlates with a lower amount of HMOs (62). Since then, several studies confirmed that pre-pregnancy BMI impacts the composition and the concentrations of HMOs during the first 4 months of lactation (67, 227, 228). Negative associations of maternal fat mass with fucosylated HMOs were also highlighted, reinforcing the role of maternal nutritional status before and during pregnancy on the composition of HMOs (172, 229). Finally, a recent interventional study using a crossover design in two different cohorts tested the effect of glucose or galactose-enriched diet for 30–57 h (*n* = 7) or a high-fat or high-carbohydrate diets for 8 days (*n* = 7) with 1–2 weeks washout between diets. Interestingly, HMO-bound fucose concentration was reduced with the glucose-enriched diet, while HMO-bound sialic acid was reduced with the high-fat diet (230). Although the cohort was relatively small and dietary intervention short, this type of clinical interventional studies with a crossover design would be insightful to fully assess the role of maternal diet upon HMO composition.

### Milk Bacteria

Factors such as geography have been shown to play a role in HM microbiota composition (144, 231, 232), although several factors may be indirectly responsible for these geographical differences, including lifestyle, environment, or diet. The relationship between maternal diet and HM microbiota was indirectly reported by Kumar et al. (231), who established a correlation between HM microbiota and specific fatty acid profiles. Likewise, HM microbiota composition was related to fatty acids, carbohydrates, and protein intake as observed by Williams et al. (102). However, the impact of maternal diet on HM bacteria deserves further investigations. Seferovic et al. in their crossover study investigating the impact of glucose or galactose on the one hand and of high-fat vs. high-carbohydrate diets in two small cohorts revealed overall minimal discernable impact of maternal diet on taxonomic composition of HM (shotgun metagenomic sequencing). However, the abundance of multiple metabolic pathways was influenced by maternal diets, including pathways involved in amino acid metabolism (230). Once again, well-powered and long-duration intervention clinical trials are warranted to further explore the role of maternal diet upon HM microbiota.

### Bacterial Metabolites

The impact of maternal diet on HM metabolites has been indirectly studied through their characterization in HM from different geographical (and therefore different diets) locations (cf. *Metabolites and Bacterial Metabolites* section) and comparing HM metabolites in lean and obese mothers (229). At 1 month postpartum, 10 HM metabolites differed between overweight/obese and lean mothers: 4/10 metabolites were nucleotide derivatives, 3/10 were HMOs, and one was a butyrate derivative (2-aminobutyrate) (229). In another study, the total polyamine content was lower at 3 days, 1, and 2 months after delivery in HM from obese mothers compared with HM from lean mothers (233). Spermine levels did not differ between groups at any time in contrast to the levels of putrescine and spermidine. The obese mothers who received dietary advice during pregnancy based on the Nordic Nutrition Recommendations had higher concentrations of putrescine and spermidine in their milk than the obese mothers without any intervention, suggesting that the low levels in obesity were at least partly associated with food habits. However, the consistency of spermine suggested a special metabolic function of this polyamine (233). Finally, a choline supplementation during the second half of gestation and the first month and a half of lactation increased HM choline and its derivatives' concentration (234).

### Hormones

As discussed earlier, HM hormones arise from maternal plasma. Thus, HM hormone concentrations are directly linked to maternal plasma concentrations (13) and thus maternal nutritional status. A direct role of maternal diet on HM hormone concentration is unknown.

## Conclusion

HM is not only a biofluid that provides the nutrients required to promote infant growth. It also contains many components whose impact on the infant gut microbiota establishment starts to be recognized. Data on causal relationships between these compounds and the infant microbiota are scarce. Current evidences rely on *in vitro* data, animal models, or association studies in humans, which highlights the need for strong convincing studies. Moreover, the amount of HM compounds reaching the colon, the role of partly digested compounds (for example, HM-derived peptides) reaching the colon, and the role of intact HM compounds on small intestine microbiota composition also need to be investigated to fully appreciate the role of HM in shaping the infant gut microbiota.

**Figure 4:**
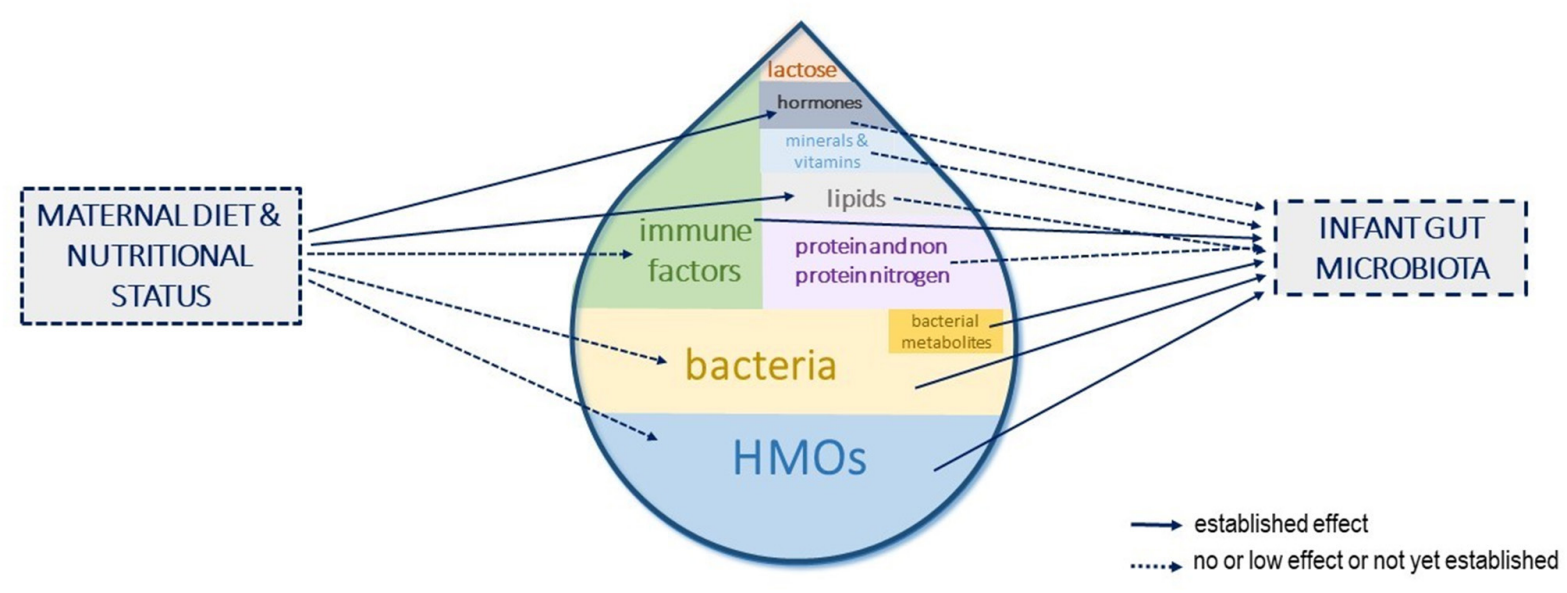
Human milk components shaping the infant gut microbiota and influence of maternal diet and nutritional status. The different components of human milk (HM) that have been shown or are suspected to influence the infant gut microbiota establishment are represented. Their size in the milk drop is proportional to their suspected role in shaping the infant gut microbiota. The influence of maternal diet or nutritional status on these HM components concentrations is depicted with arrows.

At the maternal level, a better understanding of the factors influencing compounds' concentration in HM, the interactions between them, and the persistence of the effects could open avenues to strategies to modulate the infant gut microbiota toward compositions beneficial to their health. Among the influencing factors, lifestyle and diets could be used to shape HM components toward a targeted composition that could, in turn, shape the infant gut microbiota and more largely be beneficial to infant health (Figure 4). However, studies investigating the role of maternal diet upon the main contributors to the infant gut microbiota (i.e., HMOs, bacteria, and immune factors) are still lacking. Interventional trials in large cohorts with long dietary interventions, covering both gestation and lactation and/or observational studies with well-designed frequency food questionnaires to get an in-depth characterization of mothers' eating profiles, are needed to fully understand and use the maternal diet as a leverage to shape the infant gut microbiota.

## Author Contributions

GB, EC, IL, SF-B, SL, SE, and SB wrote the manuscript. GB coordinated the review. All authors approved the final version of the manuscript.

## Conflict of Interest

The authors declare that the research was conducted in the absence of any commercial or financial relationships that could be construed as a potential conflict of interest.
